# Genetic variation in the eicosanoid pathway is associated with non-small-cell lung cancer (NSCLC) survival

**DOI:** 10.1371/journal.pone.0180471

**Published:** 2017-07-13

**Authors:** Lindsay N. Sausville, Carissa C. Jones, Melinda C. Aldrich, William J. Blot, Ambra Pozzi, Scott M. Williams

**Affiliations:** 1 Department of Genetics, Geisel School of Medicine, Dartmouth College, Hanover, NH, United States of America; 2 Department of Epidemiology and Biostatistics, Case Western Reserve University, Cleveland, OH, United States of America; 3 Vanderbilt Genetics Institute, Vanderbilt University Medical Center, Nashville, TN, United States of America; 4 Department of Thoracic Surgery, Vanderbilt University Medical Center, Nashville, TN, United States of America; 5 Division of Epidemiology, Vanderbilt University School of Medicine, Nashville, TN, United States of America; 6 International Epidemiology Institute, Rockville, MD, United States of America; 7 Department of Medicine, Vanderbilt University Medical Center, Nashville, TN, United States of America; 8 Department of Medicine, Veterans Affairs Hospital, Nashville, TN, United States of America; Southern Illinois University School of Medicine, UNITED STATES

## Abstract

Globally, lung cancer results in more deaths worldwide than any other cancer, indicating a need for better treatments. Members of the eicosanoid metabolism pathway represent promising therapeutic targets, as several enzymes involved in the generation of these lipids are dysregulated in many cancers and their inhibition reduces lung cancer growth in mouse models. However, genetic variation of enzymes involved in eicosanoid metabolism has not been adequately examined for association with lung cancer. The goal of this study was to determine whether germline genetic variation altering eicosanoid producing enzyme function and/or expression are associated with differences in lung cancer survival. We examined the association of genetic variation with mortality within eicosanoid metabolism genes in 395 non-small-cell lung cancer (NSCLC) cases from the Southern Community Cohort Study (SCCS). A total of 108 SNPs, both common and rare, in 19 genes, were examined for association. No common or rare variants were associated with lung cancer survival across the entire study population. However, rare variants in *ALOX15B* (arachidonate 15-lipoxygenase, type B) and the common variant rs12529 in *AKR1C3* (prostaglandin F synthase) were associated with NSCLC mortality in women and African Americans, respectively. Rare variants in *ALOX15B* were associated with greater mortality in women (HR = 2.10, 95% CI = 1.25–3.54, p-value = 0.005). The major allele of rs12529 in *AKCR1C3* associated with improved survival in African Americans (HR = 0.74, 95% CI = 0.59–0.92, p-value = 0.008). The lack of genetic associations among all NSCLC cases and the association among women only for rare variants in *ALOX15B* may, in part, explain the better NSCLC survival observed among women. These results raise the possibility that some subgroups within the NSCLC population may benefit from drugs targeting eicosanoid metabolism.

## Introduction

Lung cancer causes more than one million deaths annually worldwide, with most cases being non-small-cell lung cancer (NSCLC) [1–3]. A major cause of the high mortality is that most patients present with advanced-stage disease, precluding the possibility of successful surgical resection. In this case alternative treatment modalities, such as chemotherapy and radiation, are used, but they are less effective than resection of localized disease [4, 5]. The late stage of diagnosis and the aggressive nature of NSCLC together lead to a 5-year survival of only 18% and clearly point to the need for more effective therapies [5]. To target NSCLC for treatment it is necessary to better understand processes that affect tumor progression.

Broadly, tumor cells rely on the release of autocrine and paracrine factors to promote tumor growth and to alter tumor microenvironment (TME) responses. In turn, TME cells, such as endothelial cells, fibroblasts, and macrophages can secrete factors affecting tumor cell response, and this interplay regulates essential processes such as cancer cell proliferation, migration, neovascularization, and metastasis [6]. A major group of secreted factors altering these processes are the eicosanoids. These molecules are produced by either the tumor cells or TME cells, thus affecting processes such as inflammation, angiogenesis, and tumor cell growth/dissemination [7].

**Fig 1:**
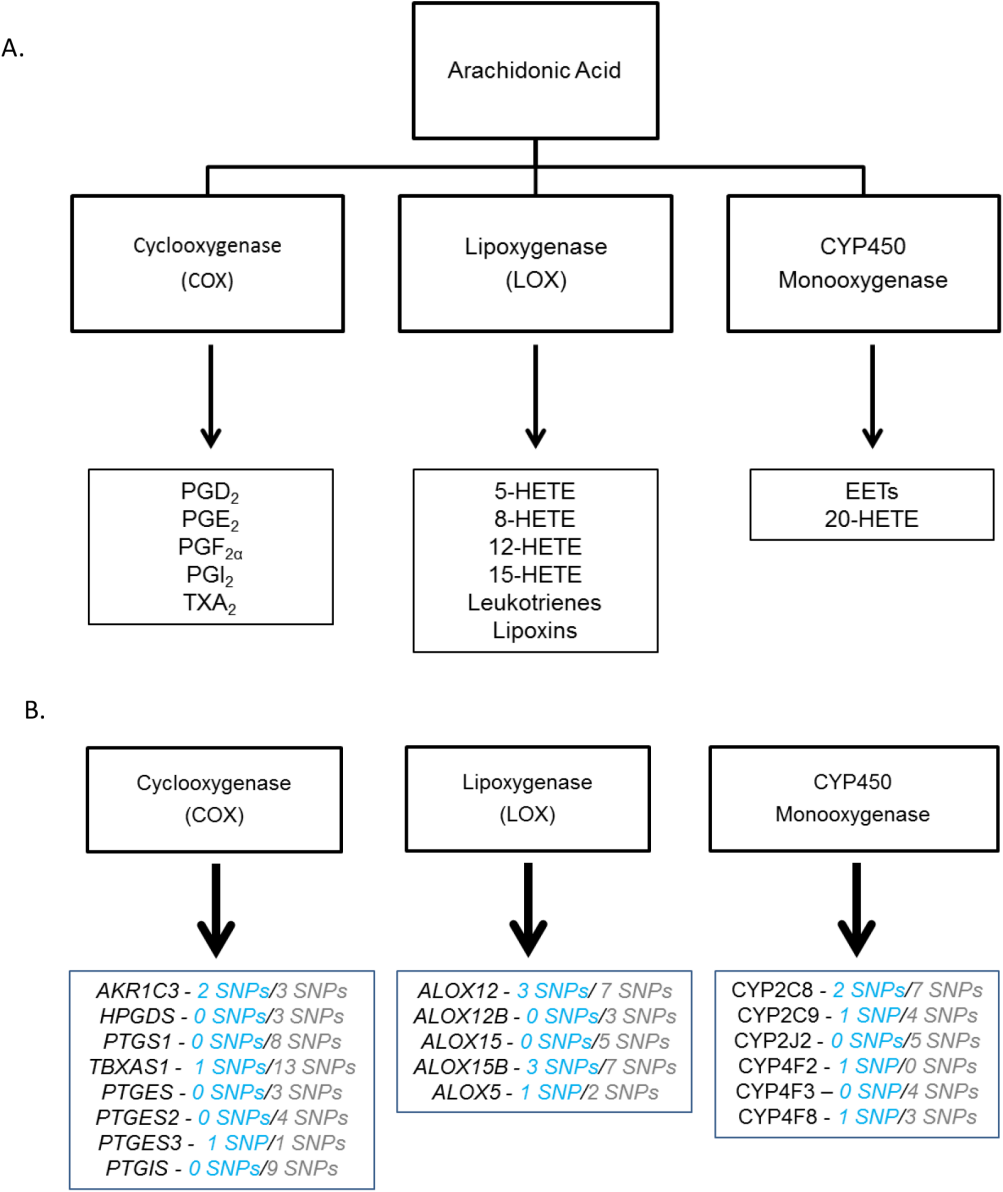
Eicosanoid metabolism. (A) Eicosanoid metabolite generation begins with the metabolism of arachidonic acid by cyclooxygenase (COX), lipooxygenase (LOX), or the cytochrome P450 monooxygenases, which includes both the cytochrome P450 epoxygenases and cytochrome p450 ω-hydroxylases. Each of these enzymes produces its own major metabolites displayed here according to the first enzyme acting on arachidonic acid. Abbreviations are as followed: prostaglandin D_2_ (PGD_2_), prostaglandin E_2_ (PGE_2_), prostaglandin F_2α_ (PGF_2α_), prostaglandin I_2_ (PGI_2_), thromboxane (TXA_2_), hydroxyeicosatetraenoic acids (HETEs), and epoxyeicosatrienoic acids (EETs). (B) The genes encoding the enzymes responsible for metabolism of major metabolites are shown here with the number of common and rare SNPs in blue and gray, respectively.

**Fig 2:**
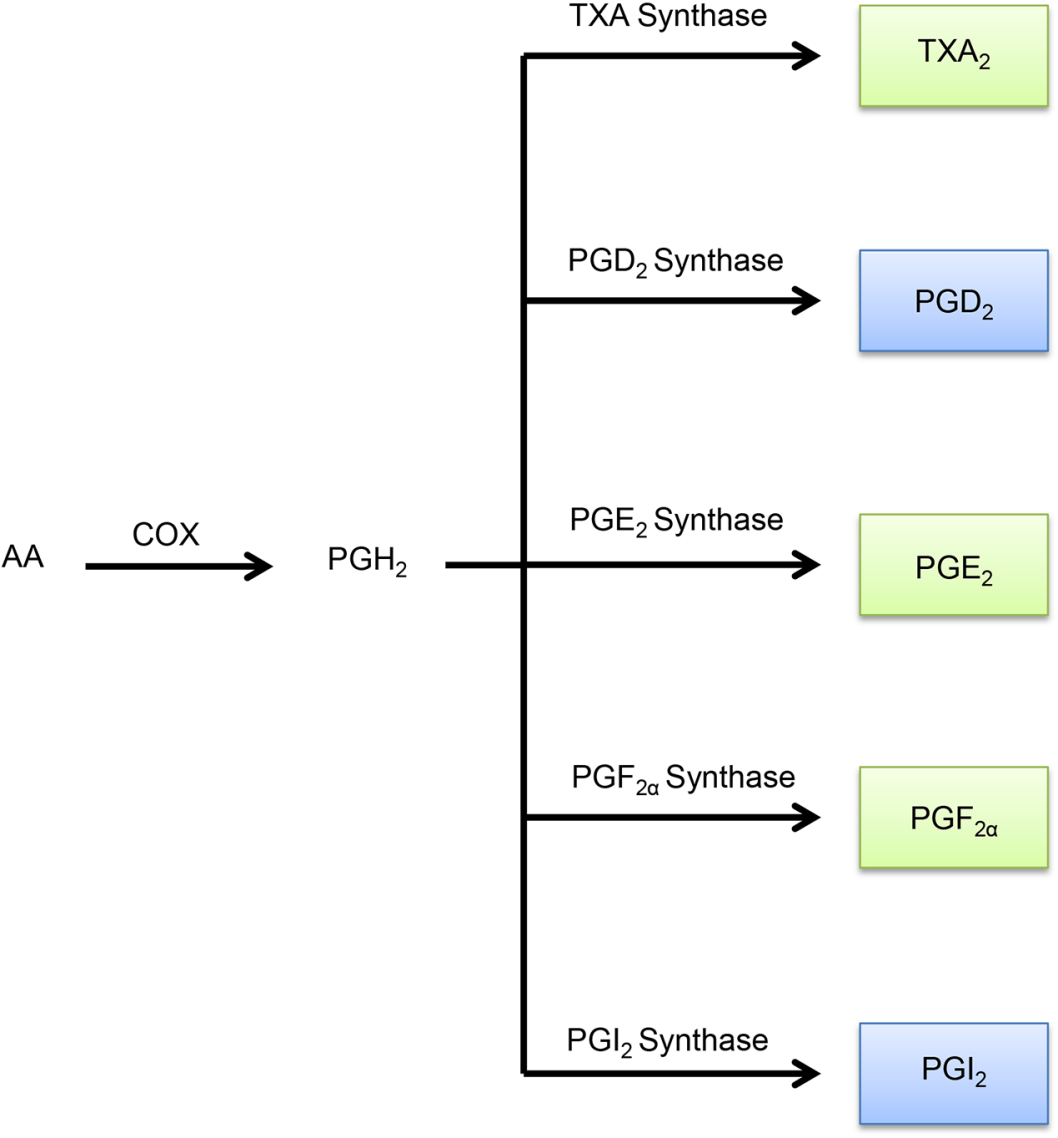
Metabolism of COX-derived eicosanoids. Arachidonic acid is first metabolized by COX-1 or COX-2 to synthesize PGH_2_. Five different types of prostaglandin synthases act on this intermediate to generate the various eicosanoids shown above. In addition, subsequent dehydration/isomerization of PGD_2_ can create 15d-PGJ_2_. With the exception of three eicosanoids, 15d-PGJ_2_, PGD_2_, and PGI_2_, all of the COX-derived eicosanoids are thought to be pro-tumorigenic. COX-derived eicosanoids in green are pro-tumorigenic, while those in blue are anti-tumorigenic. Abbreviations are as followed: 15-deoxy-Δ^12,14^-PGJ_2_ (15d-PGJ_2_), arachidonic acid (AA), prostaglandin D_2_ (PGD_2_), prostaglandin E_2_ (PGE_2_), prostaglandin F_2α_ (PGF_2α_), prostaglandin H_2_ (PGH_2_), prostaglandin I_2_ (PGI_2_), and thromboxane A_2_ (TXA_2_).

**Fig 3:**
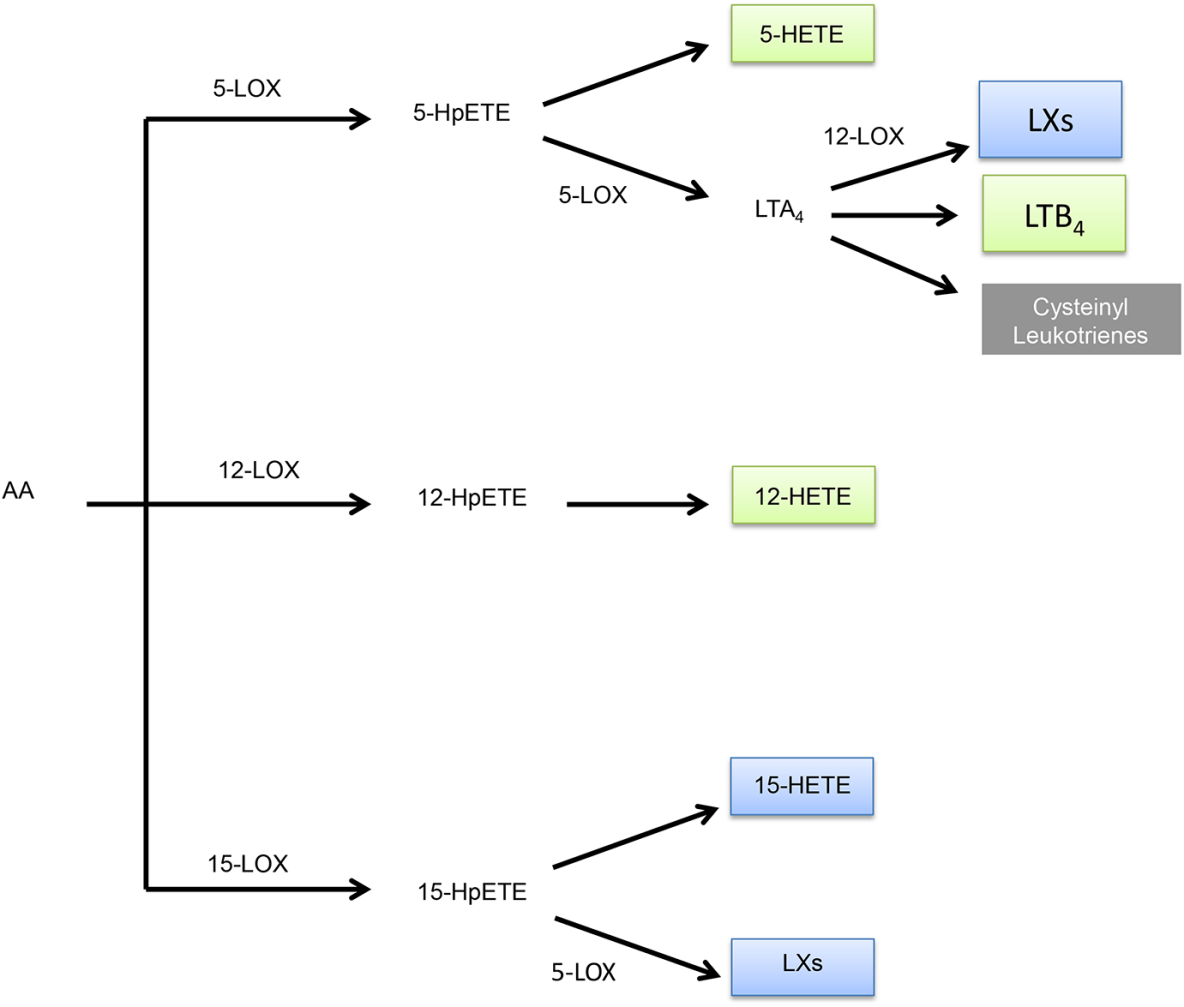
Metabolism of LOX-derived eicosanoids. The initial reaction in the LOX pathway can be carried out by 5-LOX, 12-LOX, or 15-LOX, with each LOX synthesizing its own corresponding HpETE. Subsequent reduction of these HpETEs generates HETEs. However, 5-HpETE and 15-HpETE can undergo other reactions as well. More specially, 5-LOX further metabolizes 5-HpETE to LTA_4_ that serves a precursor for LXs, LTB_4_, or cysteinyl leukotrienes. 5-LOX can also act on 15-HpETE to synthesize LXs. LOX-derived eicosanoids are color coded to indicate their effect on tumors. Blue metabolites are anti-tumorigeneic, green metabolites are pro-tumorigenic, and gray metabolites are unknown. Abbreviations are as followed: arachidonic acid (AA), hydroperoxyeicosatetraenoic acid (HpETE), hydroxyeicosatetraenoic acid (HETE), leukotriene A4 (LTA_4_), leukotriene B4 (LTB_4_), and lipoxins (LXs).

**Fig 4:**
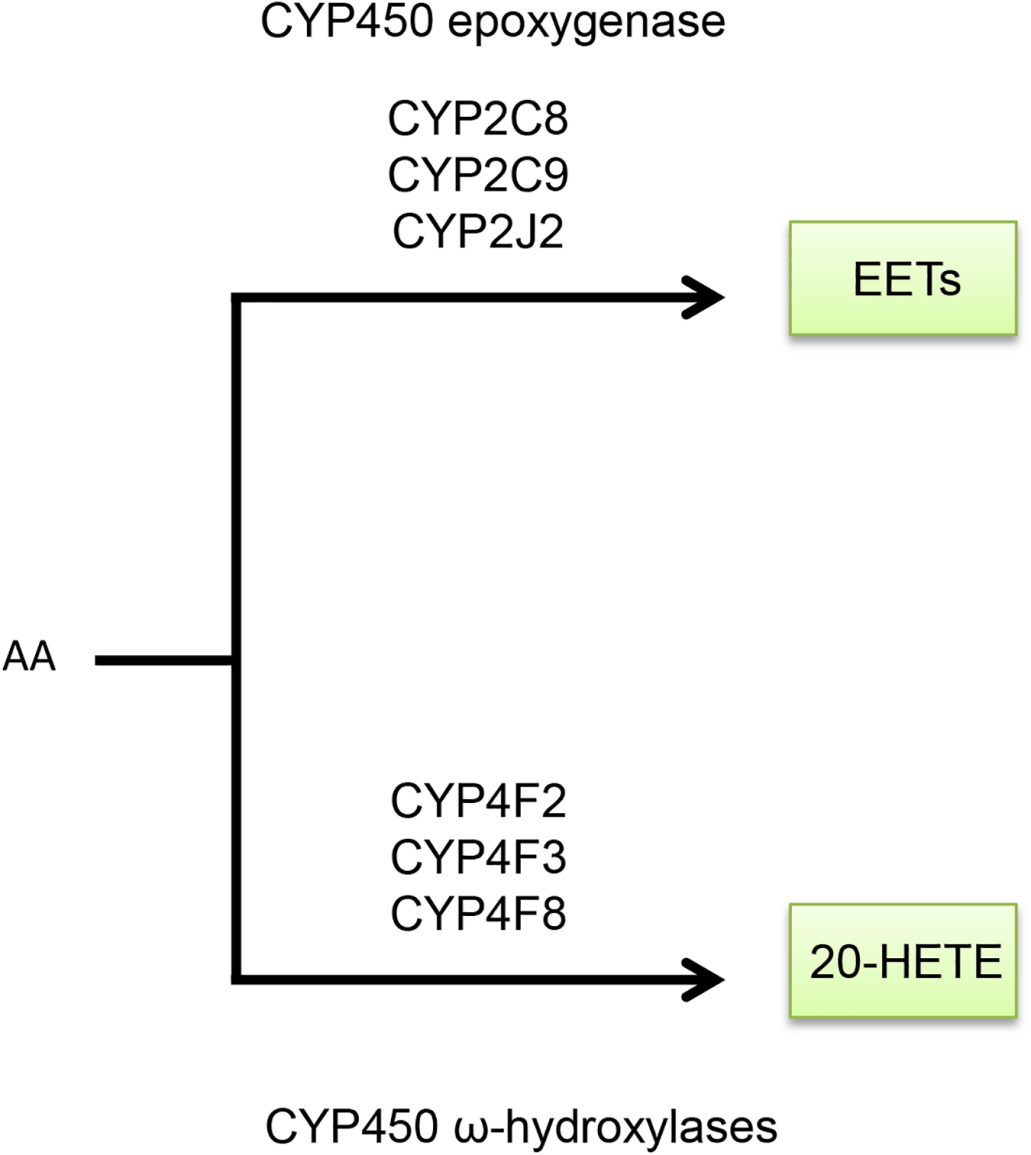
Metabolism of CYP450 monooxygenase-derived eicosanoids. Arachidonic acid can enter either of two CYP450 monooxygenase arms. The CYP450 epoxygenase arm generates EETs, while the CYP450 ω-hydroxylation arm generates 20-HETE. Abbreviations are as followed: arachidonic acid (AA), epoxyeicosatrienoic acids (EETs), and hydroxyeicosatetraenoic acid (HETE).

Eicosanoid metabolites are derived from the metabolism of arachidonic acid through three major branches: cyclooxygenase (COX), lipoxygenase (LOX), and the cytochrome P450 monooxygenase branches (Fig 1A). Each of these branches generates specific eicosanoid metabolites having different biological effects. Genes in these branches that were assayed in our study are denoted in Fig 1B. The COX-derived products begin with the generation of the prostaglandin endoperoxide prostaglandin H_2_ (PGH_2_) by COX-1 or COX-2. Selective prostaglandin synthases then convert this intermediate into corresponding prostanoids including thromboxane (TXA_2_), prostaglandin D_2_ (PGD_2_), prostaglandin E_2_ (PGE_2_), prostaglandin F_2_ (PGF_2α_), and prostaglandin I_2_ (PGI_2_) (Fig 2; S1 Table). The LOX pathway metabolizes arachidonic acid to hydro(pero)xyeicosatetraenoic acids that can undergo further reactions to generate 5-, 8-, 12-, and 15-hydroeicosatetraenoic acids (HETEs), leukotrienes, and lipoxins (Fig 3). Finally, the cytochrome P450 monooxygenases metabolize arachidonic acid to epoxyeicosatrienoic acids (EETs) or 20-HETE (Fig 4) [7].

Many of the above mentioned enzymes play a role in tumor biology based on several observations. Expression of many eicosanoid producing genes is frequently dysregulated in tumors. For example, upregulation of *COX-2* expression is common in multiple tumors types, including NSCLC, and is associated with poor prognosis by enhancing processes such as angiogenesis [8–13]. Although COX-2 inhibitors are successful in suppressing the development and progression of lung cancer in animal models, increased cardiovascular risks of COX-2 inhibitors limit their use in chemoprevention of cancer. To obviate these obstacles, blocking COX-2 activity or expression by enhancing levels of glucocorticoids, endogenous and potent COX-2 inhibitors, can suppress colon and lung cancer growth [14, 15]. In addition, natural products targeting the COX and LOX pathways have also been considered as promising treatments for cancer. In this context, curcumin, resveratrol and lycopene, to name a few, act as chemopreventive agents by targeting the COX-2 pathway; while nordihydroguaiaretic acid and baicalein play a beneficial role by inhibiting LOXs [16]. Although promising, bioavailability and possible toxicity of these natural compounds limit their use in clinics. Moreover, their beneficial effects in lung cancer have not been fully explored. In addition to COX-derived products, cytochrome P450 monooxygenase-derived products contribute to lung cancer growth and progression and genetic and/or pharmacological inhibition of these enzymes prevents lung cancer development [17–19].

If eicosanoid products play an important role in tumor progression, genetic variation altering eicosanoid producing enzyme function and/or expression may influence NSCLC survival. To address this question, we evaluated the effect of genetic variation within 20 genes that encode eicosanoid metabolism enzymes (of which only 19 had variation) on NSCLC survival in the Southern Community Cohort Study (SCCS) (Fig 1B, S1 Table). In addition, demographic features, such as race and sex, associate with NSCLC survival and may potentially influence genetic associations [5, 20–22]. Therefore, we evaluated the effect of interactions between demographic features and genetic variants in eicosanoid metabolism enzymes.

## Methods

### Characterization of cohort

The SCCS is a prospective cohort in the southeastern United States of more than 80,000 individuals that investigates chronic diseases in low income individuals, including non-small cell lung cancer (NSCLC). Participants were enrolled from March 2002 to September 2009, with most participants enrolled at community health centers. A random sample of participants from the general population was selected from voter registration records, driver license records, and commercial mailings. All participants were between the ages of 40 and 79 years at the time of study enrollment. Demographic characteristics of participants recruited at community health centers were obtained during an in-person interview, including self-reported race/ethnicity and tobacco use, while this same information was obtained through mailed questionnaires to participants from the general population. Written informed consent was obtained from all participants. Further details of the SCCS can be found elsewhere [23, 24]. This work was approved by the Vanderbilt University IRB.

Incident NSCLC cases with smoking history were identified through linkage with the 12 state cancer registries overlapping with the SCCS geographic region from 2002 to 2010. From these state cancer registries NSCLC staging and treatment information was abstracted. Staging information was based on American Joint Committee on Cancers (AJCC, 6^th^ and 7^th^ Editions) TNM System staging guidelines [25]. Stage was defined as localized, regional, and distant NSCLC disease with localized disease consisting of AJCC stage I, regional disease consisting of AJCC stages II and III, and distant disease consisting of AJCC stage IV. Treatment information was broadly captured as surgical resection, chemotherapy, or radiation. Vital status was obtained from linkages with the Social Security Administration or the National Death Index as of December 31, 2011. Survival time was defined as the time from date of NSCLC diagnosis to date of death or loss to follow-up. Since certain clinical and demographic variables, such as sex, smoking history, NSCLC stage, and NSCLC treatment may influence NSCLC outcome, the effect of these variables on mortality was examined, coding each as a discrete variable (Table 1). The Cox proportional hazards model was used to determine whether these variables were associated with mortality among non-small cell lung cancer cases in univariate models (Table 2). If a clinical or demographic variable was associated with NSCLC prognosis (p-value < 0.05), the variable was adjusted for in subsequent genetic association models.

**Table 1:** Description of clinical and demographic variables in NSCLC cases from the SCCS cohort.

Characteristic	N (%)
**Sex**	
Male	219 (55.4)
Female	176 (44.6)
**Vital Status**	
Died	333 (84.3)
Alive	62 (15.7)
**Self-reported COPD**	
Absent	330 (83.5)
Present	64 (16.2)
Missing	1 (0.3)
**Mean cigarettes per day (s.d.)**	19.2 (13.7)
Missing	5 (1.3)
**Mean age at diagnosis, years (s.d.)**	60.4 (8.8)
**NSCLC cancer stage**	
Localized	56 (14.2)
Regional	113 (28.6)
Distant	218 (55.2)
Missing	8 (2.0)
**NSCLC Histology**^a^	
Adenocarcinoma	146 (37.0)
NSCLC NOS	109 (27.6)
NSCLC Other	32 (8.1)
Squamous cell carcinoma	110 (27.9)
**Surgical resection**	
Absent	296 (74.9)
Present	74 (18.7)
Missing	25 (6.3)
**Chemotherapy**	
Absent	202 (51.1)
Present	169 (42.8)
Missing	24 (6.1)
**Radiation**	
Absent	208 (52.7)
Present	149 (37.8)
Missing	38 (9.6)

The table above depicts the distributions of clinical and demographic features in the NSCLC case. No data was missing for sex, vital status, mean age at diagnosis, or NSCLC histology. Abbreviations are as follows: chronic obstructive pulmonary disease (COPD), standard deviation (s.d.), and not otherwise specified (NOS).

^a^Histology was allowed to overlap for a given individual.

**Table 2:** Univariate association of clinical and demographic variables with mortality in NSCLC cases.

Characteristic	HR	95% CI	P-value
**Sex**			
Male	1.00	Referent	
Female	0.63	0.47–0.82	0.001
**Self-reported COPD**			
Absent	1.00	Referent	
Present	1.06	0.70–1.63	0.77
**Cigarettes per day**	1.02	1.01–1.03	<0.001
**Age at diagnosis**	1.00	0.99–1.02	0.66
**NSCLC cancer stage**			
Localized	1.00	Referent	
Regional	1.75	1.06–2.90	0.029
Distant	3.48	2.18–5.54	<0.001
**NSCLC Histology**^**a**^			
Adenocarcinoma	1.00	Referent	
NSCLC NOS	1.27	0.92–1.76	0.15
NSCLC Other	1.15	0.68–1.94	0.61
Squamous cell carcinoma	1.11	0.80–1.54	0.54
**Surgical resection**			
Absent	1.00	Referent	
Present	0.25	0.16–0.39	<0.001
**Chemotherapy**			
Absent	1.00	Referent	
Present	0.93	0.71–1.21	0.59
**Radiation**			
Absent	1.00	Referent	
Present	0.97	0.74–1.27	0.82

The association of clinical and demographic variables with survival was examined with univariate Cox proportional hazard models.

^a^If a NSCLC case had more than one histology, histology was set to missing for survival analysis.

### Genotype information

NSCLC cases were genotyped on the Illumina HumanExome BeadChip v1.1. Quality control measures at the individual level excluded any individual with sex inconsistencies or genotyping efficiency less than 98%. Using PLINK, individuals were examined for genetic relatedness; in this sample one pair of relatives was identified and the individual with higher genotyping efficiency was kept in the sample [26]. Based on these criteria our study population consisted of 395 incident NSCLC cases with 265 African Americans and 130 European Americans. SNP level quality control removed SNPs on the sex chromosomes and SNPs < 98% genotyping efficiency.

We extracted genetic information on 20 eicosanoid metabolism genes and examined 108 variants in these genes for association with mortality in NSCLC (Fig 1B, S1 Table). Genomic locations were obtained from the NCBI Gene ID for each gene in GRCh37.p5, and single nucleotide polymorphisms (SNPs) corresponding to the genomic location of each gene were extracted from the larger exome data. For analysis, SNP classification was based on the minor allele frequency (MAF) with rare defined in our study as less than or equal to 10% and common greater than 10%. A relatively high MAF was chosen to define SNPs as rare due to the small sample size. Common and rare SNPs were analyzed independently of each other using different statistical methods.

### Common genetic variant analysis

As common SNPs often rely on linkage disequilibrium (LD) with causative variants and LD patterns differ with race/ethnicity, common SNP analyses were stratified by race [27, 28]. The Kaplan-Meier estimator was used to determine which common SNPs were nominally associated with NSCLC mortality (p-value < 0.1). For all survival analyses, censoring occurred at time of death, loss to follow-up, or at the end of the follow-up period (December 31, 2011). As the Kaplan-Meier estimator is a non-parametric statistic, it was used initially to identify genetic variants for further analyses [29]. A Cox proportional hazard model was used to assess the genetic associations for nominally associated SNPs, adjusted for the variables noted above that were significantly associated with prognosis. The effect of common genetic variation was measured using an additive genetic model, except when fewer than 5 homozygotes for the minor allele were present. In this situation, a dominant model was used where the rare homozygotes for the minor allele were combined with the heterozygote individuals. SNP-sex interactions were assessed in African Americans to determine when appropriate to stratify by sex (p-value < 0.05). However, European American SNP-sex interactions were not evaluated due to their small sample size and thus none of these SNP associations were sex stratified. To correct for multiple testing, a false discovery rate (FDR) procedure was used with a FDR of 15% based on the 16 SNPs [30].

### Rare genetic variant analysis

Assessment of the effect of rare SNPs on lung cancer survival occurred at the gene-level using the Combined Multivariate and Collapsing (CMC) method [31]. In brief, the CMC method collapses rare genetic variants within a single gene into a binary variable for association analysis [31]. This gene-level variable indicates the presence or absence of rare SNPs within the gene. Since most of the rare SNPs on the genotyping platform used reside in exons, only exonic SNPs composed the gene-level variable. Genes with fewer than 5 individuals possessing a rare SNP within that gene were excluded from analysis (*CYP4F2*).

We used the Kaplan-Meier estimator to determine the association of the rare gene-level variable with NSCLC survival. Genes associated with NSCLC survival at a p-value < 0.1 were further examined using Cox proportional hazard models; one model was unadjusted, while the other model was adjusted for variables significant in preliminary demographic analyses. To determine when appropriate to stratify by sex for genetic associations, the significance of an interaction term between the collapsed genetic variants and sex was examined in a Cox proportional hazards model. When the p-value was less than 0.05, downstream analyses were conducted stratified by sex. We assessed race and SNP interactions in the same manner to determine when appropriate to stratify by race. Multiple testing was corrected with a FDR of 15% based on the 18 genes tested.

### Generation of principal components

Despite examining race-specific genetic interactions for rare variants and stratifying by self-reported race, we cannot exclude the possibility of confounding by residual ancestral population differences in our sample population. To account for any residual ancestral population differences, we generated the first three principal components (PCs) for all individuals using the entire NSCLC population and genome-wide SNPs in EIGENSTRAT [32]. The effect of SNPs significantly associated with lung cancer was calculated in an adjusted model that included other prognostic variables and the first three PCs.

Putative functional effects of SNPs were determined using SIFT, which predicts the effect of amino acid substitutions based on conservation across species [33]. LD plots were generated using r^2^ values for all SNPs within a given gene using Haploview software [34]. STATA 12.0 (College Station, Texas) was used for all statistical analyses.

## Results

From the years of 2002 to 2010, a total of 395 incident cases of NSCLC with smoking history were diagnosed in the SCCS cohort (Table 1). At presentation of disease, 55.2% of the NSCLC cases were diagnosed with distant stage disease. Histology was predominately adenocarcinoma (37.0%), followed by squamous cell carcinoma and NSCLC not otherwise specified (27.9% and 27.6%, respectively). Treatment for NSCLC broadly consisted of surgical resection, chemotherapy, and radiation. Distribution of treatments reflects the restriction of a given treatment to certain stages of NSCLC with no single treatment received by the majority of the population. For example, surgical resection is typically limited to local and regional stage disease. For individuals who underwent the above treatments a combination of chemotherapy and radiation was the most common treatment (21.3%), which is consistent with the frequency of advanced stage disease in this population. Of the NSCLC cases, 22% did not receive any of these three types of treatments. Finally, approximately 15% of the individuals were missing information for one or more treatment variables (Table 3).

**Table 3:** Description of combination of treatment variables.

Treatment(s)	N (%)
**No treatment**	87 (22.0)
**Surgical resection only**	47 (11.9)
**Chemotherapy only**	49(12.4)
**Radiation only**	45(11.4)
**Surgical resection + chemotherapy**	11 (2.8)
**Surgical resection + radiation**	6 (1.5)
**Chemotherapy + radiation**	84 (21.3)
**Surgical resection + chemotherapy + radiation**	8 (2.0)
**One or more treatment variable(s) missing**	58 (14.7)

The distribution of combinations of treatments is depicted above. Note that the no treatment category does not reflect other treatments outside of surgical resection, chemotherapy, and radiation.

Clinical and demographic variables were examined for association with NSCLC mortality (Table 2). Female sex and surgical resection were associated with improved outcome, while increased cigarette consumption was associated with increased mortality. In addition, NSCLC cancer stage associated with survival, with distant disease exhibiting the greatest mortality. The four significantly associated variables, sex, surgical resection, cigarettes per day, and NSCLC stage were included as covariates in all adjusted Cox models.

A total of 108 SNPs in 19 genes were available for analysis (Fig 1B and S1 Table). Common SNP analyses included 16 SNPs in 10 genes (MAF ≥ 0.10) (Fig 1B). After restriction to exonic SNPs, 92 rare SNPs in 18 genes remained for gene-level analyses (Fig 1B). Most of these genes contained between 1 and 5 rare variants within the collapsed variable, with *TBXAS1* having the most variants with 13 (Fig 1B). In addition, the COX pathway branch contained the majority of the SNPs with a total of 48 common and rare SNPs (Fig 1B). Of the 19 included genes, only 9 genes were included in both common and rare genetic variant analysis (Fig 1B).

### A common SNP in the prostaglandin F synthase (*AKR1C3*) associates with differences in NSCLC survival in African Americans

Common eicosanoid metabolism gene SNP analyses were stratified by race to account for underlying LD pattern and MAF differences. In European Americans, a total of 12 SNPs in 9 genes and in African Americans a total of 14 SNPs in 9 genes were examined. Of these SNPs, 10 overlapped between the two ethnicities; 2 SNPs were examined exclusively in European Americans and 4 SNPs were examined exclusively in African Americans. SNP-sex interactions were characterized in the African Americans to determine if differential effects were present in the two sexes and if stratification by sex was appropriate for further analyses. Two SNPs, rs2105450 (*AKR1C3*, prostaglandin F synthase) and rs4792147 (*ALOX15B*, arachidonate 15-lipoxygenase, type B) had significant SNP-sex interactions with p-values < 0.05 (Table 4), but neither of these SNPs were significantly associated with NSCLC survival in multivariable models among men and women. (S2A–S2D Fig and S2B Table).

**Table 4:** Common SNPs interaction with sex in African Americans unadjusted for covariates, N = 265.

rsID	Gene	P value
rs12529	*AKR1C3*	0.64
rs2105450	*AKR1C3*	0.01
rs1126667	*ALOX12*	0.64
rs434473	*ALOX12*	0.72
rs9895916	*ALOX15B*	NA
rs4792147	*ALOX15B*	0.03
rs7225107	*ALOX15B*	NA
rs2228064	*ALOX5*	0.15
rs11572081	*CYP2C8*	NA
rs1934951	*CYP2C8*	0.36
rs4086116	*CYP2C9*	NA
rs2108622	*CYP4F2*	0.68
rs2958154	*PTGES3*	0.45
rs3801150	*TBXAS1*	0.77

Interactions between common SNPs and sex are reported above. P-values for the sex-SNP interaction term were determined in models including SNP, sex and SNP-sex interactions only. When the interaction between sex and the SNP was significant at a p-value < 0.05, all analyses of these SNPs was conducted stratified by gender.

**Fig 5:**
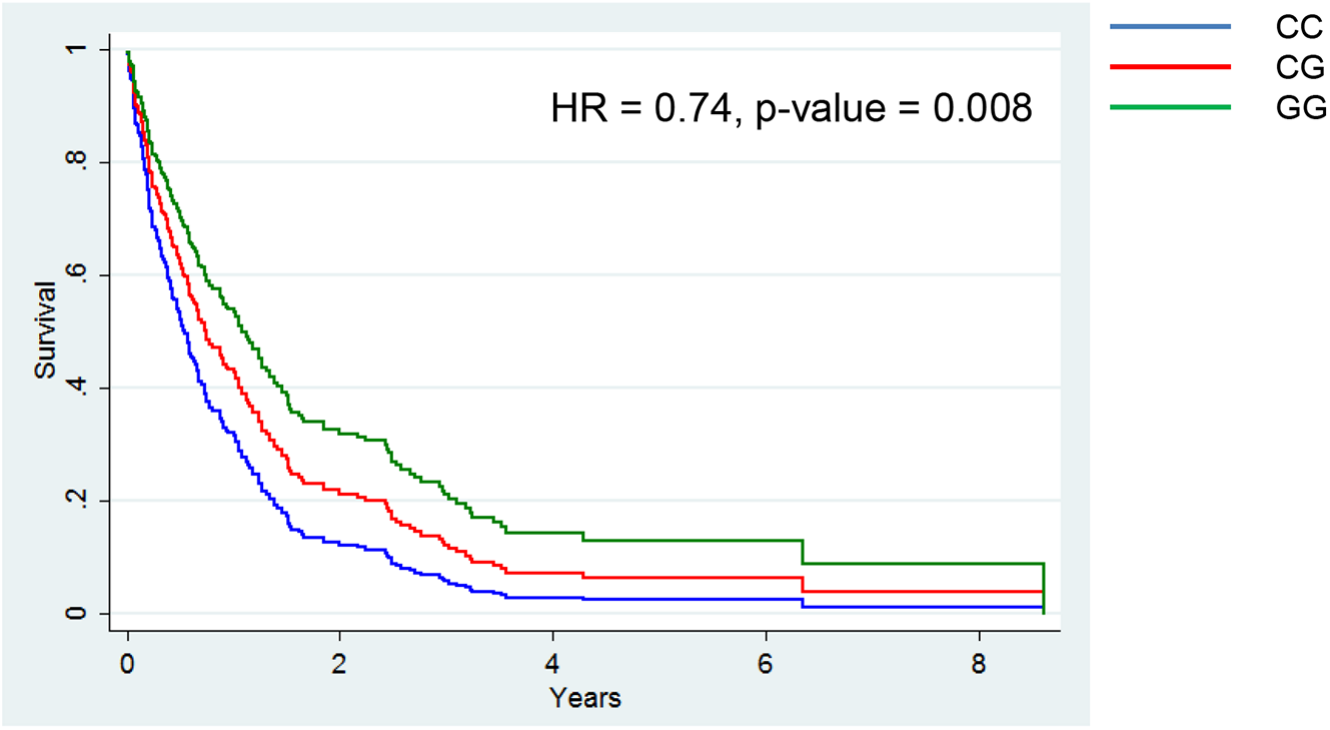
Association of a common *AKR1C3* SNP, rs12529, with NSCLC survival in African Americans, (N = 265). Survival curves for each genotypic class are plotted based on the multivariate Cox proportional model, which included adjustment for sex, cigarettes per day, resection, and NSCLC stage.

A single SNP in *AKR1C3*, rs12529, was associated with NSCLC outcome in African Americans (S3 Table). When adjusting for other prognostic variables, the minor allele of rs12529 (G) remained associated with reduced mortality in African Americans, meaning each additional copy of minor allele for this SNP improved NSCLC survival further (HR = 0.74, 95% CI = 0.59–0.92, p = 0.008) (Table 5 and Fig 5). Adjustment for the first three PCs in addition to the other prognostic variables, revealed this SNP remained significantly associated (p-value = 0.014) (S3 Fig). Of note, rs12529 was not associated with mortality in the European American NSCLC cases despite a MAF greater than 40%, indicating the lack of association is likely not the result of low MAF (S3 and S4 Tables).

**Table 5:** Association of common variants adjusted for prognostic variables.

						95% CI		
SNP	Gene	Alleles (Major/Minor)	MAF	Race/Ethnicity	HR^1^	Lower	Upper	P-value
rs12529	*AKR1C3*	C/G	47.7%	African American	0.74	0.59	0.92	0.008
rs11571340	*ALOX12*	A/G	10.0%	European American	0.76	0.43	1.33	0.34
rs9895916	*ALOX15B*	G/A	1.9%	European American	1.92	0.68	5.47	0.22
rs7225107	*ALOX15B*	A/G	19.6%	African American	0.88	0.69	1.13	0.32

Common SNPs associated with prognosis using Cox proportion hazard models. Association with NSCLC mortality was examined adjusted for sex, cigarettes per day, surgical resection, and disease stage. Minor allele frequency was calculated in the appropriate race/ethnicity. For rs12529, N = 265, for rs11571340, N = 130, for rs9895916, N = 130, and for rs7225107, N = 265. Abbreviations are as followed: MAF is minor allele frequency.

^1^ Defined relative to the major allele.

In addition, one SNP in *ALOX15B*, rs7225107, was associated with NSCLC prognosis in African Americans, while another *ALOX15B* SNP, rs9895916, was associated in European Americans (S3 Table). When adjusted for other prognostic variables, neither *ALOX15B* SNP remained significantly associated with mortality within each race (Table 5). It is noted that *ALOX15B* SNPs, both rare and common, had extremely low or no LD in both European Americans and African Americans in our sample, so the association of these common SNPs is likely independent from the other genotyped markers (S4 Fig). This pattern of LD is consistent with data from existing databases such as HapMap [27, 28].

### Rare exonic variants in *ALOX15B* Associate with NSCLC prognosis in women

Rare exonic variants in eicosanoid metabolism genes were tested for association with NSCLC survival. Prior to determining the genetic association, the statistical significance of both gene-sex and gene-race interactions was examined to determine when stratifying by sex and race, respectively, was appropriate for evaluating the genetic association. Two of these interactions were significant: the interaction between *ALOX15B* and sex and *TBXAS1* and race (S5 Table). However, rare variants in *TBXAS1* did not associate with NSCLC survival in either race stratified.

**Fig 6:**
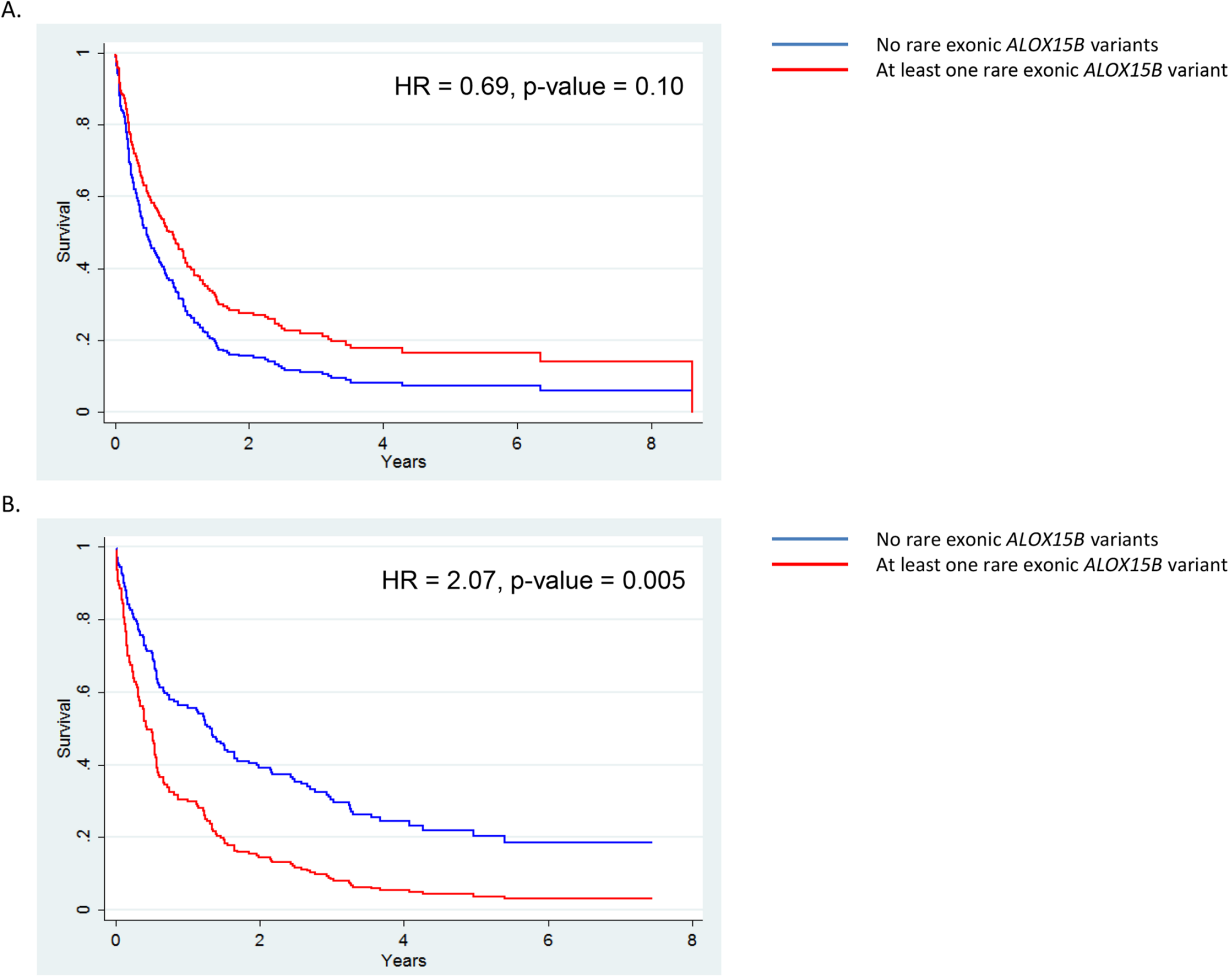
Rare Exonic *ALOX15B* SNPs associate with a worse prognosis in female NSCLC patients. Sex-stratified survival curves calculated from the corresponding sex-specific univariate Cox proportional hazard model are shown above with the hazard ratio and p-values reported from the Cox proportional hazard model. The blue survival line is individuals without any rare *ALOX15B* variants and the red survival line is individuals with rare *ALOX15B* variants. (A) The survival curve and association of *ALOX15B* SNPs in men (N = 217), and (B) the survival curve and association in women (N = 175).

**Fig 7:**
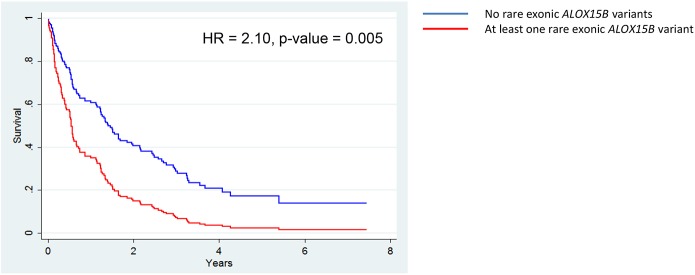
Rare *ALOX15B* SNPs associate with worse prognosis in adjusted analysis, (N = 175). Multiple variable adjusted survival curves for women were generated from an adjusted Cox proportional model. Variables adjusted for include resection, cigarettes per day, and NSCLC staging. Both the hazard ratio and p-value from the Cox proportional hazard model are reported.

Initial associations examined using the Kaplan-Meier estimator indicated that none of the rare variants in eicosanoid metabolism genes were associated with differences in NSCLC survival, but the presence of the interaction between *ALOX15B* and sex suggested the effect of rare variants in *ALOX15B* on prognosis differs by sex (S5 Table). Sex-stratified analyses showed rare *ALOX15B* exonic variants were associated with decreased survival in men, but associated with increased survival in women, although the association was only statistically significant among women (Fig 6). To further characterize this association in women, the association of *ALOX15B* variants was adjusted for surgical resection, cigarettes per day, and NSCLC staging. The association of *ALOX15B* variants remained statistically significant in the adjusted model (HR = 2.10, 95% CI = 1.25–3.54, p-value = 0.005) (Fig 7). Inclusion of the first three PCs, in addition to the other prognostic variables, did not appreciably alter this association (HR = 2.07) (S5 Fig). Finally, although rare exonic variants in *ALOX12* and *PTGIS* were associated with NSCLC mortality (p-value < 0.05) using the log-rank test these associations were not statistically significant in adjusted models (S6 and S7 Tables).

## Discussion

The goal of this study was to determine whether exonic variation in the eicosanoid pathway is associated with NSCLC survival. To do this, we investigated the association of genetic variation in eicosanoid metabolism genes with NSCLC prognosis in the Southern Community Cohort Study. No genetic variants were significantly associated with NSCLC mortality. The lack of an association for the entire population is not unexpected since prognosis differs by sex. However, variation within *ALOX15B* and the prostaglandin F synthase *AKR1C3* associated significantly with NSCLC survival in certain subgroups of the NSCLC cases. We found that rare exonic *ALOX15B* variants were associated with poorer survival among women with NSCLC and rs12529 in *AKR1C3* was associated with better prognosis in African Americans with NSCLC. Despite the decreased survival in women with rare ALOX15B variants, overall women still survive longer than men. This may be because the SNPs we studied were too rare to affect outcome averaged across all women. Although this may also be due to women being more proactive in their treatment, data indicates that under equal treatment women still survive better than men indicating some still unknown factors impacting survival [35–37].

Our results show an independent association between sex and survival with female NSCLC cases surviving longer than men with the same disease. This is consistent with prior studies also demonstrating improved NSCLC survival in women [35–37]. Some studies indicate that better NSCLC survival in women is limited to or more pronounced in cases with adenocarcinoma and this histology is more common in women, but other studies have failed to replicate these findings [35–39] The reasons for the differential survival between men and women are not well understood; however, one study suggested that tumors from women with NSCLC are enriched for changes in expression of immunity-related genes despite having similar composition of immune cells to their male counterparts [40]. Along with our own findings, these studies indicate that NSCLC may have important biological differences between the two sexes that drive survival differences.

ALOX15B, or 15-LOX-2, is a lipoxygenase enzyme that metabolizes arachidonic acid to produce 15(S)-HETE. ALOX15B has been proposed as a tumor suppressor gene, as its major product 15(S)-HETE inhibits prostate cancer cell cycle progression and VEGF synthesis [41–43]. In contrast to these studies, 15(S)-HETE promotes angiogenesis in adipose tissues and stimulates VEGF production in endothelial cells derived from adipose tissues, suggesting that this lipid mediator can either prevent or promote angiogenesis and that its effects are cell type and tissue specific [44]. The role of *ALOX15B* in lung cancer biology is poorly characterized. Although this enzyme is expressed in both normal and cancerous lung tissues, *ALOX15B* expression correlates inversely with both NSCLC cancer cell proliferation and NSCLC cancer grade [45, 46]. The inverse correlation between ALOX15B levels and lung cancer grade can be explained by the fact that 15(S)-HETE can bind and activate PPARγ, a nuclear transcription factor shown to inhibit the growth of human NSCLC cells as well as prevent smoking-induced lung cancer development in mice [45, 47, 48]. Interestingly, half of the associated rare exonic *ALOX15B* SNPs we have identified are predicted to be loss-of-function by SIFT (S8 Table). Thus, the worse prognosis in NSCLC individuals is consistent with the idea that ALOX15B-derived 15(S)-HETE plays a protective role in the course of lung cancer either by activating PPARγ or by downregulating the expression of pro-angiogenic genes.

AKR1C3 acts on a large number of substrates including steroid hormones, prostaglandins, and proximate carcinogens of polycyclic aromatic hydrocarbons (PAHs) [49–55]. Among its enzymatic activity AKR1C3 generates PGF_2_ epimers that bind and activate prostaglandin F receptors [56, 57]. Increased PGF_2α_ production in endometrial cancer promotes progression by enhancing angiogenesis and proliferation [58–61]. The potential role of PGF_2_ epimers in NSCLC biology is not well characterized, but given their role in promoting endometrial tumorigenesis it is certainly plausible these molecules have similar effects in NSCLC, and prior genetic studies have identified AKR1C3 as associating with bladder and lung cancer risk [62, 63]. Here we found that a common variant in *AKR1C3*, rs12529, associated with improved NSCLC prognosis in African Americans. The failure to find a comparable trend in the European descent populations cannot be explained at the present, especially as we do not know the exact nature of the causal relationship even in African Americans.

Mechanistically, the observed association of rs12529 may be explained several ways. The associated SNP may in itself be causative, but it should be noted that while rs12529 is located within the coding region of *AKR1C3* SIFT predicts the SNP as benign and no studies have characterized this SNP as functional to date. In addition, although AKR1C3 possesses prostaglandin-F synthase activity, its ability to act on other classes of substrate cannot be excluded as a mechanistic explanation for the observed association with NSCLC survival. Another explanation for this association is that rs12529 associates indirectly with NSCLC prognosis, meaning rs12529 is in LD with a causative genetic variation rather than being causative itself. Further characterization of genetic variation in AKR1C3 will be necessary to distinguish these possibilities.

Despite the two associations found above, there are limitations to our study. First, the sample size of this study was relatively small, especially when examining associations among strata. It is likely that even if these associations are real that the effect size is inaccurate creating a need to replicate these findings to better estimate the effect size. However, replication was not attempted here due to the scarcity of an appropriate population for replication. Another limitation was the use of the CMC method for analysis of rare variants in the event the rare variants have opposite directions of effect, biasing the association towards the null. In addition, the CMC method cannot capture differences in effect size among rare variants. As a result the association of collapsed rare variants represents an average effect size that may misestimate the real effects [31].

The association of arachidonic acid genetic variation with NSCLC survival suggests NSCLC cases with certain genotypes may benefit from treatments targeting eicosanoid metabolism, and that patient attributes, such as genotype, sex, and race, may also influence NSCLC treatment decisions. We found multiple sex-SNP interactions that influence NSCLC survival, where sex determined the direction of effect for a SNP. On this basis, treatments targeting eicosanoid metabolism will likely have sex-specific differences. However, we cannot rule out the role of sex-specific NSCLC histology differences in driving sex-SNP interactions due to inadequate sample size. Despite these limitations, the association of rare variants in *ALOX15B* in women may begin to explain the observed differences in NSCLC survival between men and women. Overall, these findings still suggest a more personalized approach in NSCLC treatment would benefit patients and targeting eicosanoid metabolism may be a viable NSCLC treatment in certain patients.

In conclusion, we have identified two arachidonic acid metabolizing enzymes whose genetic variation associated significantly with NSCLC survival. Their effect appears to differ by sex and race, indicating that different approaches to treatment may be necessary as a result of either. Therefore, it is possible that eicosanoid metabolism interventions may be used to slow NSCLC progression. Finally, since the pathways we have studied have existing drugs to treat cardiovascular disease, a number of drugs already exist that may be potentially re-purposed for the treatment of NSCLC.

## Supporting information

S1 Fig Statistical analyses workflow. (TIF)

S2 Fig Common SNPs in arachidonic acid metabolism genes interact with sex to influence African American NSCLC progression (TIF)

S3 Fig The association of rs12529 with NSCLC survival in African Americans, n = 265. (TIF)

S4 Fig *ALOX15B* SNPs have little LD. (TIF)

S5 Fig Rare *ALOX15B* variants associate with worse prognosis when adjusted for variables including top 3 PCs, n = 175. (TIF)

S1 Table Arachidonic acid metabolism pathway genes. (DOCX)

S2 Table Association of SNPs interacting with sex in African Americans. (DOCX)

S3 Table Association of common SNPs within eicosanoid metabolism genes. (DOCX)

S4 Table Comparison of minor allele frequencies in *AKR1C3* SNPs in European and African Americans. (DOCX)

S5 Table Interaction of demographic variables with rare genetic variants in NSCLC survival association, n = 395. (DOCX)

S6 Table Association of rare SNPs collapsed by gene with NSCLC survival, n = 395. (DOCX)

S7 Table Univariate and multivariate associations of rare SNPs. (DOCX)

S8 Table SIFT predictions for rare *ALOX15B* variants. (DOCX)
