# Supplementary figures and images for: Genetic variation in the eicosanoid pathway is associated with non-small-cell lung cancer (NSCLC) survival
Source: PLoS One. 2017 Jul 13;12(7):e0180471. doi: 10.1371/journal.pone.0180471 (PMC5509150; doi:10.1371/journal.pone.0180471)

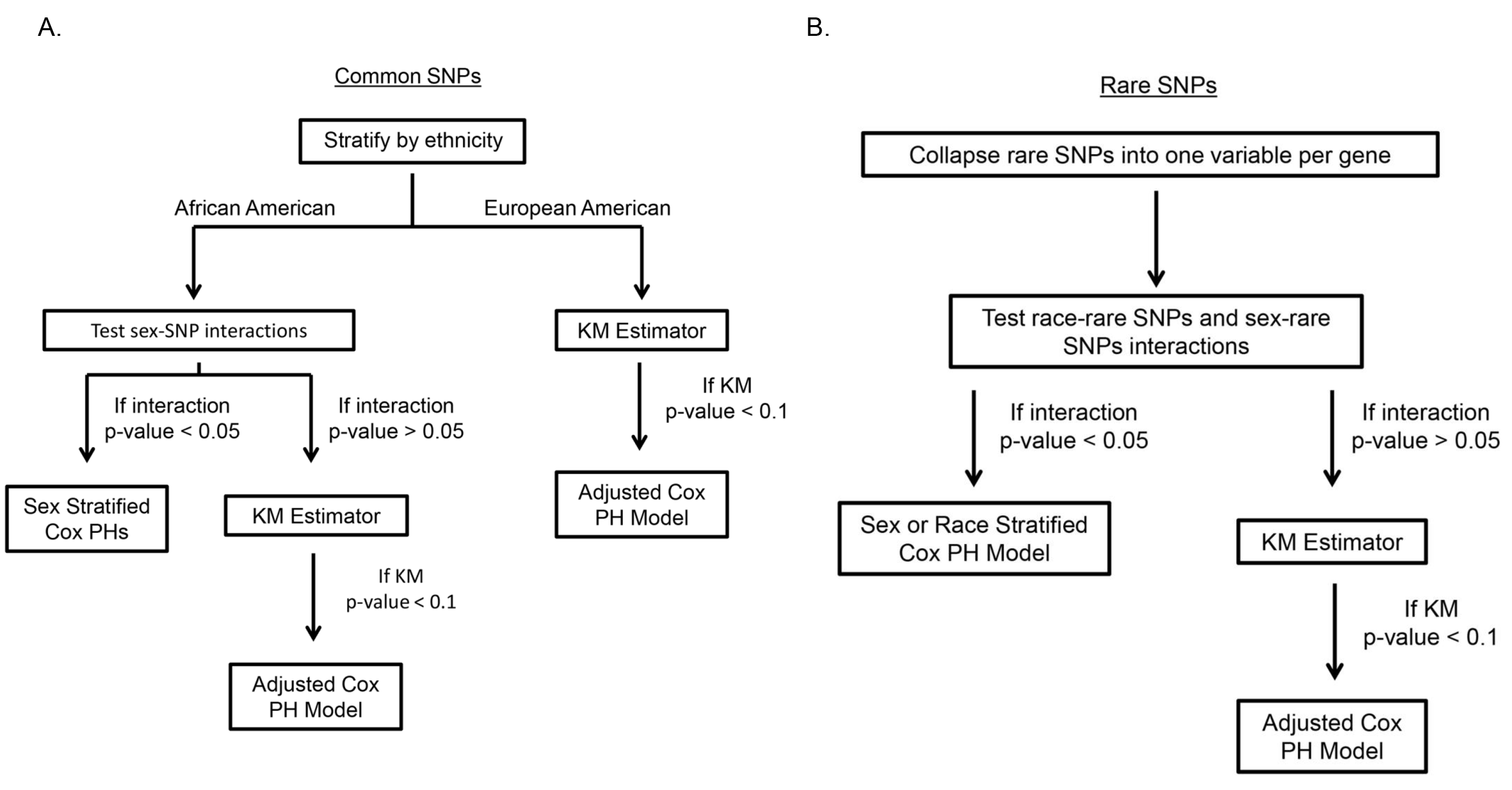

Supplement: S1 Fig — (TIF) [file pone.0180471.s001.tif]

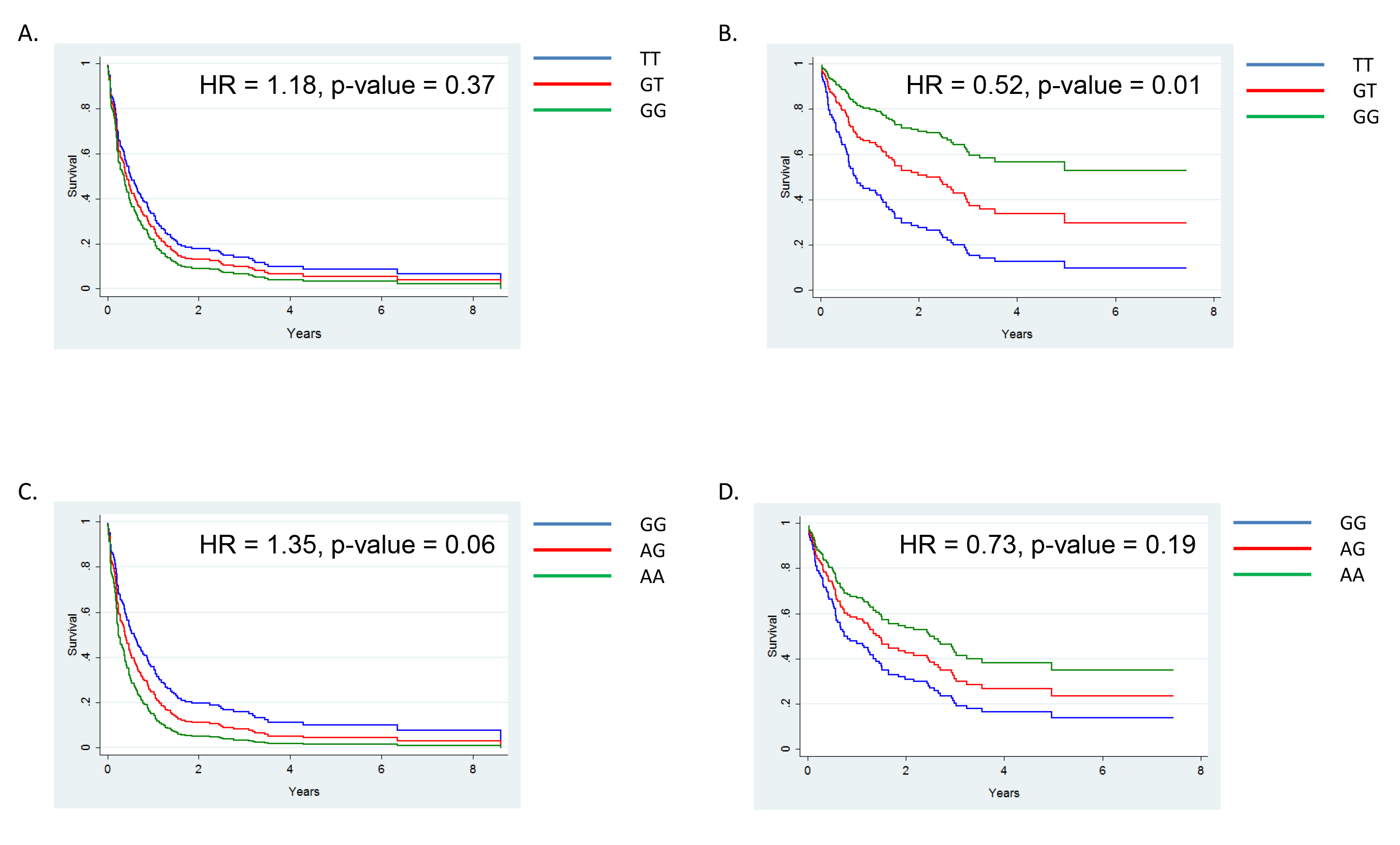

Supplement: S2 Fig — (TIF) [file pone.0180471.s002.tif]

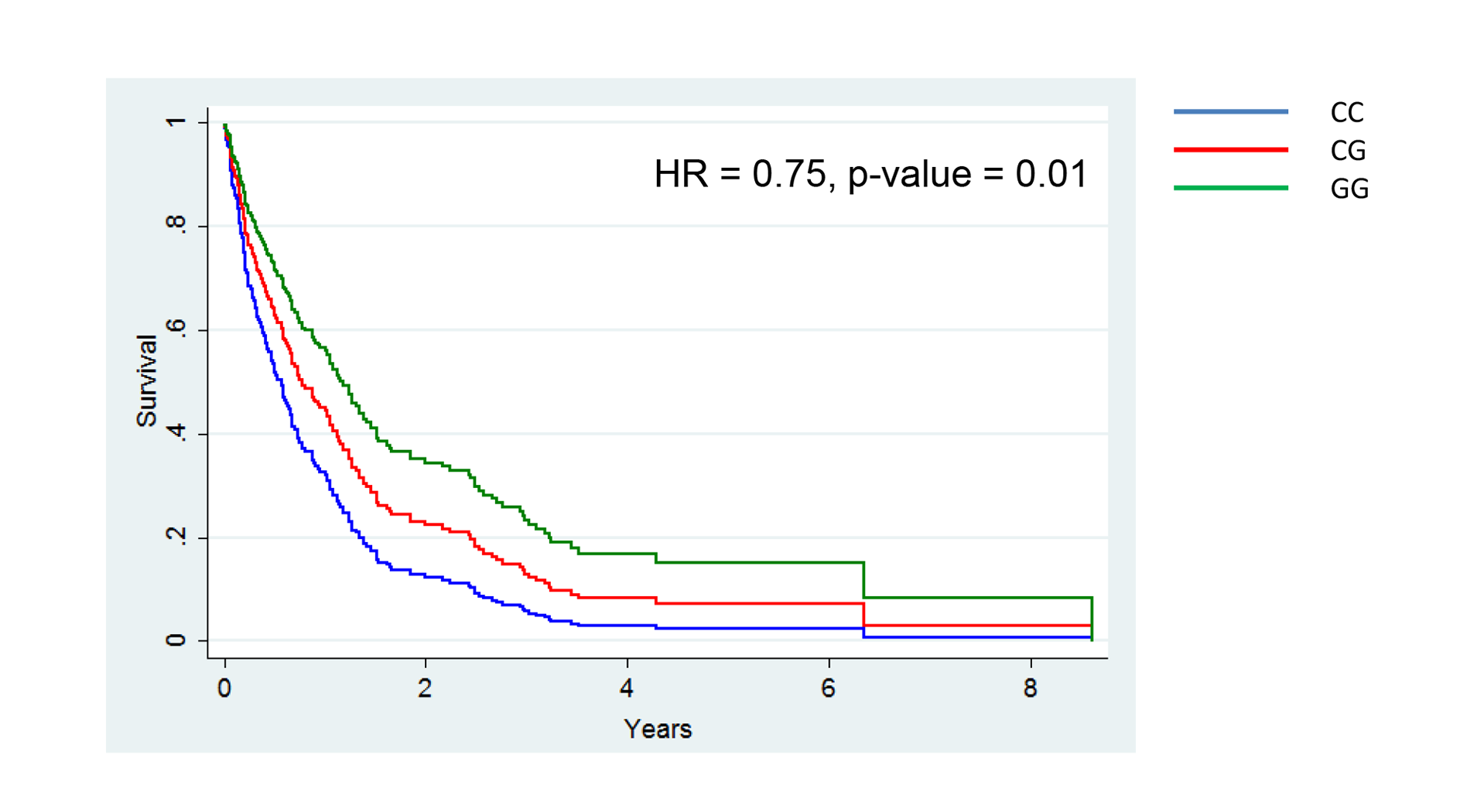

Supplement: S3 Fig — (TIF) [file pone.0180471.s003.tif]

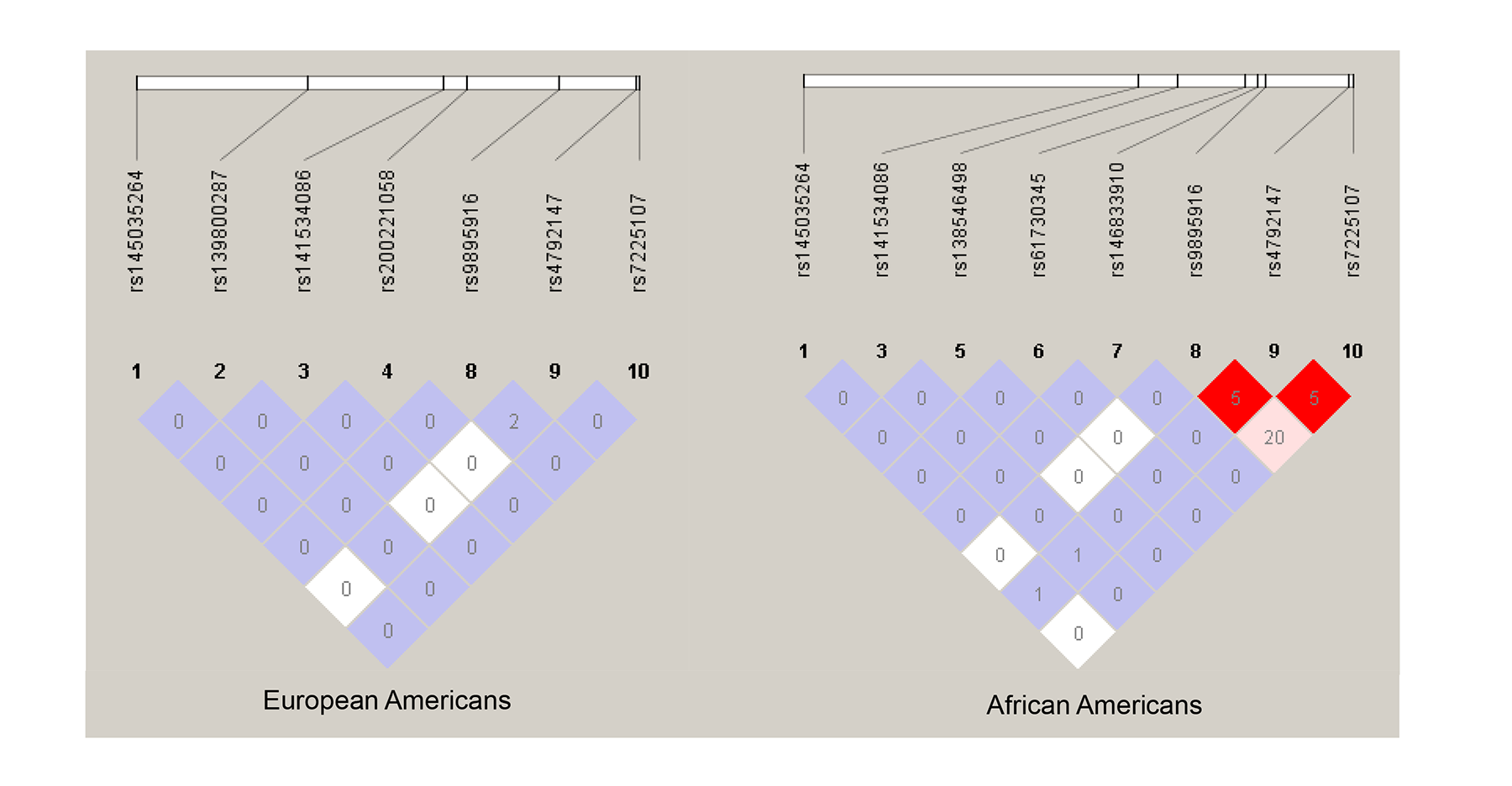

Supplement: S4 Fig — (TIF) [file pone.0180471.s004.tif]

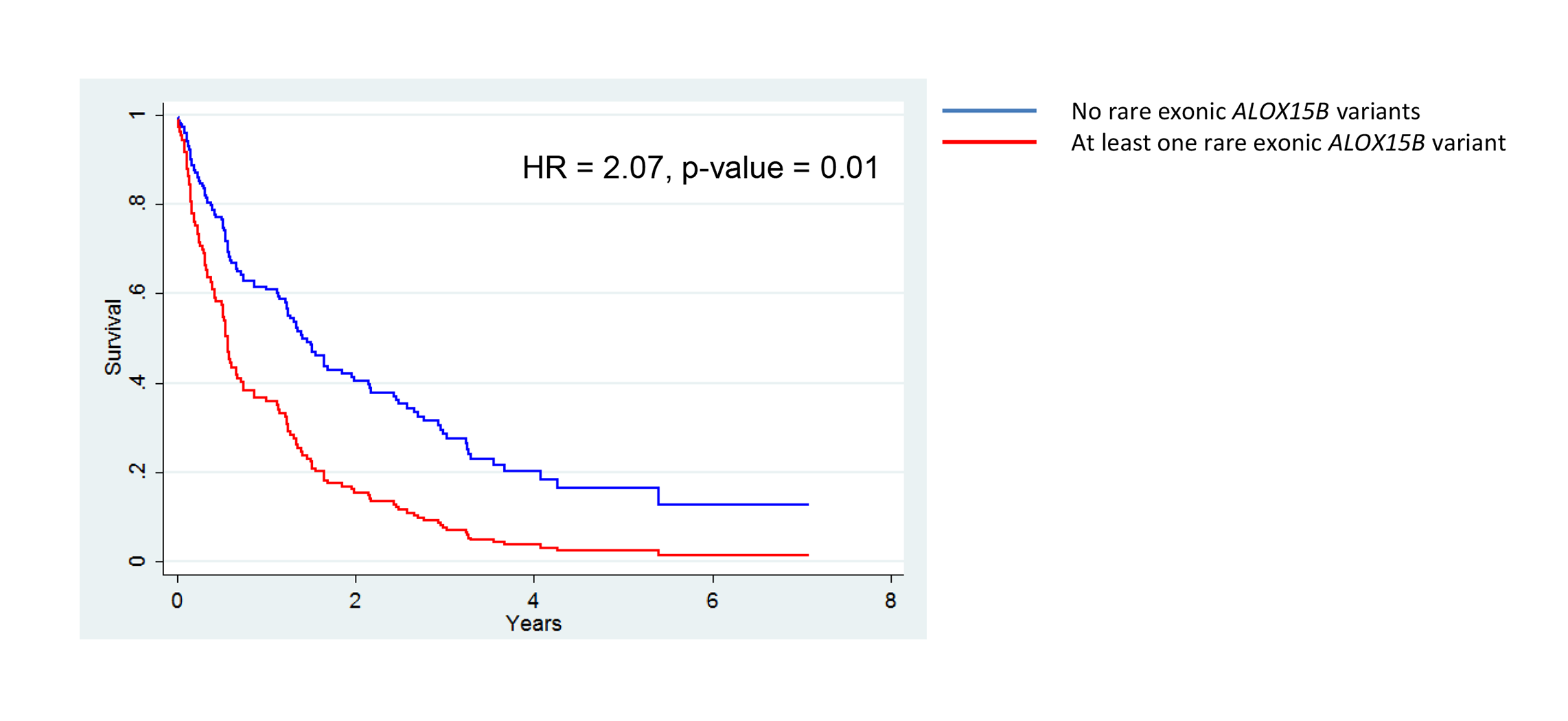

Supplement: S5 Fig — (TIF) [file pone.0180471.s005.tif]
