# Supplementary material for: Genetic variation in the eicosanoid pathway is associated with non-small-cell lung cancer (NSCLC) survival
Source: PLoS One. 2017 Jul 13;12(7):e0180471. doi: 10.1371/journal.pone.0180471 (PMC5509150; doi:10.1371/journal.pone.0180471)
Supplement: S1 Table — (DOCX) [file pone.0180471.s006.docx]

**S1 Table. Arachidonic acid metabolism pathway genes.**

| **Gene** | **Enzyme** | **Also Known As** |
| --- | --- | --- |
| *AKR1C3* | Prostaglandin F Synthase | DD3, DDX, PGFS, HSD17B5 |
| *ALOX12* | 12-Lipoxygenase | LOG12, 12-LOX, 12S-LOX |
| *ALOX12B* | 12-Lipoxygenase | ARCI2, 12R-LOX |
| *ALOX15* | 12- and 15-Lipoxygenase | 12-LOX, 15-LOX-1 |
| *ALOX15B* | 15-Lipoxygenase | 15-LOX-2 |
| *ALOX5* | 5-Lipoxygenase | 5-LO, 5LPG, LOG5, 5-LOX |
| *CYP2C8* | CYP450 Epoxygenase | CPC8, CYPIIC8 |
| *CYP2C9* | CYP450 Epoxygenase | CPC9, CYP2C, CYP2C10, CYPIIC9 |
| *CYP2J2* | CYP450 Epoxygenase | CPJ2, CYPIIJ2 |
| *CYP4F2* | CYP450 ω-Hydroxylase | CPF2 |
| *CYP4F3* | CYP450 ω-Hydroxylase | CPF3, CYP4F, LTB4H |
| *CYP4F8* | CYP450 ω-Hydroxylase | CPF8, CYPIVF8 |
| *HPGDS* | Prostaglandin D Synthase | PGD2, PGDS |
| *PGTES* | Prostaglandin E Synthase | PGES, mPGES-1 |
| *PTGES2* | Prostaglandin E Synthase | PGES2, mPGES-2 |
| *PTGES3* | Prostaglandin E Synthase | cPGES |
| *PTGIS* | Prostaglandin I Synthase | PGIS, CYP8A1 |
| *PTGS1* | Prostaglandin G/H Synthase | COX-1, COX1 |
| *PTGS2* | Prostaglandin G/H Synthase | COX-2, COX2 |
| *TBXAS1* | Thromboxane Synthase | TS, TXAS, CYP5A1 |

A total of 20 genes were included from the arachidonic acid metabolism pathway. All genes except *PTGS2* contained polymorphic SNPs allowing for evaluating the effects of common and/or rare genetic variation on NSCLC survival.
