# Supplementary material for: Genetic variation in the eicosanoid pathway is associated with non-small-cell lung cancer (NSCLC) survival
Source: PLoS One. 2017 Jul 13;12(7):e0180471. doi: 10.1371/journal.pone.0180471 (PMC5509150; doi:10.1371/journal.pone.0180471)
Supplement: S2 Table — (DOCX) [file pone.0180471.s007.docx]

**S2A-B Table. Association of SNPs interacting with sex in African Americans**

**A)**

|  | **Men** | | | | **Women** | | | |
| --- | --- | --- | --- | --- | --- | --- | --- | --- |
|  |  | **95% CI** | |  |  | **95% CI** | |  |
| **SNP** | **HR** | **Lower** | **Upper** | **P-value** | **HR** | **Lower** | **Upper** | **P-value** |
| rs2105450 | 1.18 | 0.83 | 1.68 | 0.37 | 0.52 | 0.32 | 0.87 | 0.01 |
| rs4792147 | 1.35 | 0.98 | 1.86 | 0.06 | 0.73 | 0.46 | 1.17 | 0.19 |

**B)**

|  | **Men** | | | | **Women** | | | |
| --- | --- | --- | --- | --- | --- | --- | --- | --- |
|  |  | **95% CI** | |  |  | **95% CI** | |  |
| **SNP** | **HR** | **Lower** | **Upper** | **P-value** | **HR** | **Lower** | **Upper** | **P-value** |
| rs2105450 | 1.26 | 0.87 | 1.85 | 0.23 | 0.62 | 0.35 | 1.09 | 0.10 |
| rs4792147 | 1.27 | 0.89 | 1.80 | 0.18 | 0.77 | 0.47 | 1.26 | 0.29 |

The association of the two SNPs with significant sex interactions was determined stratified by sex. Both an (A) unadjusted and (B) adjusted Cox proportional hazard model was used. The multivariate association was adjusted for cigarettes per day, resection, and NSCLC staging. For rs2105450, N = 163 for men and N = 101 for women and for rs4792147, N = 163 for men and N = 102 for women.
