# Supplementary material for: Genetic variation in the eicosanoid pathway is associated with non-small-cell lung cancer (NSCLC) survival
Source: PLoS One. 2017 Jul 13;12(7):e0180471. doi: 10.1371/journal.pone.0180471 (PMC5509150; doi:10.1371/journal.pone.0180471)
Supplement: S3 Table — (DOCX) [file pone.0180471.s008.docx]

**S3 Table. Association of Common SNPs** **within Eicosanoid Metabolism Genes**

|  |  | **Kaplan Meier p-value** | |
| --- | --- | --- | --- |
| **rsID** | **Gene** | **European American** | **African American** |
| rs12529 | *AKR1C3* | 0.97 | 0.08 |
| rs2105450 | *AKR1C3* | 0.34 | 0.30 |
| rs1126667 | *ALOX12* | 0.80 | 0.97 |
| rs434473 | *ALOX12* | NA | 0.65 |
| rs11571340 | *ALOX12* | 0.07 | NA |
| rs9895916 | *ALOX15B* | 0.008 | 0.50 |
| rs4792147 | *ALOX15B* | 0.87 | 0.78 |
| rs7225107 | *ALOX15B* | NA | 0.04 |
| rs2228064 | *ALOX5* | NA | 0.38 |
| rs11572081 | *CYP2C8* | NA | 0.37 |
| rs1934951 | *CYP2C8* | 0.43 | 0.93 |
| rs4086116 | *CYP2C9* | 0.23 | 0.76 |
| rs2108622 | *CYP4F2* | 0.51 | 0.50 |
| rs2056822 | *CYP4F8* | 0.96 | NA |
| rs2958154 | *PTGES3* | 0.21 | 0.57 |
| rs3801150 | *TBXAS1* | 0.99 | 0.45 |

The effect of common SNPs on NSCLC survival was evaluated with the Kaplan Meier estimator with p-values within each race reported above. SNPs with nominal associations (p-value > 0.1) were included in further analyses in the appropriate race.
