# Supplementary material for: Genetic variation in the eicosanoid pathway is associated with non-small-cell lung cancer (NSCLC) survival
Source: PLoS One. 2017 Jul 13;12(7):e0180471. doi: 10.1371/journal.pone.0180471 (PMC5509150; doi:10.1371/journal.pone.0180471)
Supplement: S4 Table — (DOCX) [file pone.0180471.s009.docx]

**S4 Table. Comparison of minor allele frequencies in AKR1C3 SNPs in European and African Americans.**

| **SNP** | **European American MAF** | **African American MAF** |
| --- | --- | --- |
| rs2105450 | 0.250 | 0.133 |
| rs12529 | 0.408 | 0.477 |
| rs34186955 | 0.012 | 0 |
| rs146552480 | 0 | 0.004 |
| rs9643 | 0 | 0.006 |

Minor allele frequency of *AKR1C3* SNPs was calculated within each race separately**.**
