# Supplementary material for: Genetic variation in the eicosanoid pathway is associated with non-small-cell lung cancer (NSCLC) survival
Source: PLoS One. 2017 Jul 13;12(7):e0180471. doi: 10.1371/journal.pone.0180471 (PMC5509150; doi:10.1371/journal.pone.0180471)
Supplement: S5 Table — (DOCX) [file pone.0180471.s010.docx]

**S5 Table. Interaction of demographic variables with rare genetic variants in NSCLC survival association, N = 395.**

|  | **Interaction p-values** | |
| --- | --- | --- |
| **Gene** | **Sex** | **Race** |
| *AKR1C3* | NA | NA |
| *ALOX12* | 0.95 | NA |
| *ALOX12B* | NA | 0.89 |
| *ALOX15* | 0.29 | 0.65 |
| *ALOX15B* | 0.001 | NA |
| *ALOX5* | 0.96 | NA |
| *CYP2C8* | 0.46 | 0.57 |
| *CYP2C9* | 0.46 | 0.94 |
| *CYP2J2* | 0.72 | NA |
| *CYP4F3* | NA | NA |
| *CYP4F8* | NA | NA |
| *HPDGS* | 0.72 | NA |
| *PTGES* | 0.96 | NA |
| *PTGES2* | 0.23 | 0.40 |
| *PTGES3* | NA | NA |
| *PTGIS* | 0.42 | NA |
| *PTGS1* | 0.13 | 0.22 |
| *TBXAS1* | 0.08 | 0.04 |

Interactions between sex and race with the collapsed rare genetic variant binary variable were evaluated. When there were not sufficient numbers of individuals with rare SNPs in a gene the p-value of the interaction was not reported. If the interaction was significant (p-value < 0.05), stratified analysis by the appropriate variable was conducted.
