# Supplementary material for: Genetic variation in the eicosanoid pathway is associated with non-small-cell lung cancer (NSCLC) survival
Source: PLoS One. 2017 Jul 13;12(7):e0180471. doi: 10.1371/journal.pone.0180471 (PMC5509150; doi:10.1371/journal.pone.0180471)
Supplement: S6 Table — (DOCX) [file pone.0180471.s011.docx]

**S6 Table. Association of rare SNPs collapsed by gene with NSCLC survival, N = 395.**

| **Gene** | **P-value** |
| --- | --- |
| *AKR1C3* | 0.59 |
| *ALOX12* | 0.04 |
| *ALOX12B* | 0.69 |
| *ALOX15* | 0.53 |
| *ALOX5* | 0.71 |
| *ALOX15B* | 0.58 |
| *CYP2C8* | 0.33 |
| *CYP2C9* | 0.15 |
| *CYP2J2* | 0.65 |
| *CYP4F3* | 0.88 |
| *CYP4F8* | 0.64 |
| *HPDGS* | 0.20 |
| *PTGES* | 0.96 |
| *PTGES2* | 0.38 |
| *PTGES3* | 0.78 |
| *PTGIS* | 0.10 |
| *PTGS1* | 0.39 |
| *TBXAS1* | 0.81 |

The effect of the collapsed rare variants was reported for each gene. Genetic associations were calculated using the Kaplan Meier estimator.
