# Supplementary material for: Genetic variation in the eicosanoid pathway is associated with non-small-cell lung cancer (NSCLC) survival
Source: PLoS One. 2017 Jul 13;12(7):e0180471. doi: 10.1371/journal.pone.0180471 (PMC5509150; doi:10.1371/journal.pone.0180471)
Supplement: S7 Table — (DOCX) [file pone.0180471.s012.docx]

**S7 Table. Univariate and multivariate associations of rare SNPs**

|  | **Unadjusted** | | | | **Adjusted** | | | |
| --- | --- | --- | --- | --- | --- | --- | --- | --- |
|  |  | **95% CI** | |  |  | **95% CI** | |  |
| **Gene** | **HR** | **Lower** | **Upper** | **P-value** | **HR** | **Lower** | **Upper** | **P-value** |
| *ALOX12* | 1.54 | 1.02 | 2.32 | 0.04 | 1.31 | 0.85 | 2.03 | 0.22 |
| *PTGIS* | 1.40 | 0.93 | 2.11 | 0.11 | 1.23 | 0.81 | 1.88 | 0.33 |

The association of genes with rare variants with significant associations (p-value < 0.05) in the Kaplan-Meier estimator was further characterized using Cox proportional hazard models. The adjusted models included adjustments for sex, resection, cigarettes per day, and NSCLC staging. A total of 395 individuals had complete genotype information on all SNPs were available for each of the two genes.
