# Supplementary material for: Genetic variation in the eicosanoid pathway is associated with non-small-cell lung cancer (NSCLC) survival
Source: PLoS One. 2017 Jul 13;12(7):e0180471. doi: 10.1371/journal.pone.0180471 (PMC5509150; doi:10.1371/journal.pone.0180471)
Supplement: S8 Table — (DOCX) [file pone.0180471.s013.docx]

**S8 Table. SIFT predictions for rare *ALOX15B* variants.**

| **SNP** | **Predicted Effect** |
| --- | --- |
| rs145035264 | Tolerated |
| rs139800287 | Damaging |
| rs141534086 | Damaging |
| rs200221058 | Not Found |
| rs138546498 | Damaging |
| rs61730345 | Tolerated |
| rs146833910 | Tolerated |

The effect of each rare *ALOX15B* SNP was predicted using SIFT.
